# Cationic Emulsion Polymerization of Octamethylcyclotetrasiloxane (D4) in Mixtures with Alkoxysilanes

**DOI:** 10.3390/molecules27030605

**Published:** 2022-01-18

**Authors:** Janusz Kozakiewicz, Joanna Trzaskowska, Michał Kędzierski, Joanna Sołtysiak, Elżbieta U. Stolarczyk, Izabela Ofat-Kawalec, Jarosław Przybylski

**Affiliations:** Łukasiewicz Research Network—Industrial Chemistry Institute, Rydygiera 8, 02-724 Warsaw, Poland; joanna.trzaskowska@ichp.pl (J.T.); michal.kedzierski@ichp.pl (M.K.); joanna.soltysiak@ichp.pl (J.S.); elzbieta.stolarczyk@ichp.pl (E.U.S.); izabela.ofat-kawalec@ichp.pl (I.O.-K.); jaroslaw.przybylski@ichp.pl (J.P.)

**Keywords:** octamethylcyclotetrasiloxane, alkoxysilanes, DBSA, emulsion polymerization, starved feed, FTIR ATR

## Abstract

The cationic emulsion polymerization of octamethylcyclotetrasiloxane (D4) in mixtures with methyltriethoxysilane (MTES) and vinyltriethoxysilane (VTES) was studied by FTIR ATR, GC, the development of a toluene insoluble fraction of the polymer and a gravimetric analysis. The polymerization of D4 alone was also conducted for comparison and, additionally, the development of molecular weight of polydimethylsiloxane (PDMS) obtained in that process was studied by GPC. Dodecylbenzenesulphonic acid (DBSA) was used as a surfactant and catalyst. The process was carried out in a “starved feed” mode by adding dropwise the monomer mixture to the aqueous solution of DBSA. FTIR ATR spectra were recorded by the sensor placed in the probe tip of a ReactIR 15^TM^ apparatus. It was found that the silicone polymer formation proceeded faster when D4 was polymerized in the mixture with alkoxysilanes, especially in the beginning of the process, and that already at the beginning of the process, the partly crosslinked polymer was formed. The induction period of ca. 30 min was observed and the concentration of cyclic siloxanes (D4 and decamethylcyclopentasiloxane—D5) remained at a very low level in the course of the reaction and only traces were detected in the final product. The particle size development in the course of the reaction was also studied and it was found that the particle size distribution was bimodal and was broadening as the reaction proceeded, though this phenomenon was less distinct when D4 was polymerized in the mixtures with alkoxysilanes. The structure of the reaction product was confirmed by ^29^Si NMR.

## 1. Introduction

**Figure 1:**
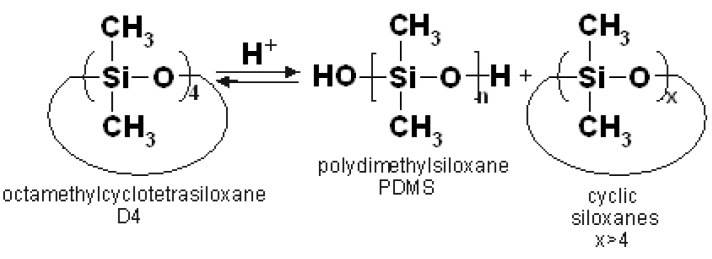
Cationic polymerization of octamethylcycloterasiloxane (D4).

The cationic polymerization of octamethylcyclotetrasiloxane (D4) catalysed by acids leads to the formation of polydimethylsiloxane (PDMS), though higher molecular weight cyclosiloxanes are also formed as side products (Figure 1) [1,2,3,4].

The emulsion polymerization of D4, first described in 1959 [5], is preferred over polymerization carried out in bulk because, usually, the monomer conversion is higher, the polymer molecular weight (MW) distribution is lower and less cyclic oligomers of a higher MW than D4 are formed [6]. When dodecylbenzenesulfonic acid (DBSA) is applied as a surfactant and at the same time as a polymerization initiator, the cationic emulsion polymerization proceeds smoothly at a relatively low temperature [1,3,4,6,7,8,9].

First, results of studies on the effect of selected parameters of the emulsion polymerization of D4 carried out in the presence of DBSA were published in 1969 [10]. It was proved that the monomer conversion increased when the DBSA concentration increased. However, the results of later studies on the emulsion copolymerization of D4 with tetramethyltetravinylcyclotetrasiloxane (ViD_4_) showed that such a statement was true only up to a certain concentration of DBSA, namely, 0.27% [11] or 1.84 × 10^−2^ mol/dm^3^, i.e., about 0.6% [12]. A three-layer interface model explaining the role of DBSA in the process of the emulsion polymerization of D4 in the presence of DBSA was also provided in the same paper [12]. According to that model, DBSA molecules were thought to locate in the middle layer at the interface.

The increase in monomer conversion with an increase in DBSA concentration in the reaction mixture was later confirmed and the effect of other parameters on that conversion was investigated [13]. It was proved that the monomer conversion may be distinctly influenced by the use of other surfactants along with DBSA, as well as by the concentration and rate of addition of D4 and by the stirring rate. A significant effect of temperature on monomer conversion that had been first detected earlier [10] was also confirmed. As it was shown in the other study, the monomer conversion could be enhanced by decreasing the size of monomer droplets because of the increase in the overall surface at the D4/water interphase, where the ring opening reaction proceeded [11].

The effect of various factors on the polymer molecular weight (MW) and particle size in the emulsion polymerization of D4 in the presence of DBSA was also investigated [6,8,9,13,14,15,16]. It was reported that MW increased when the reaction temperature decreased [6,10], and that there was an optimal concentration of DBSA in the reaction mixture (2%) that resulted in the highest MW of the polymer [14]. It was also shown that the particle size of the polymer increased when the size of monomer droplets increased [11], and it was proved that the latter depended on the DBSA concentration [14,15]. The other factors that were found to influence the particle size were the use of co-surfactants [13] and the way D4 was introduced into the reaction mixture [14,16]. The effect of various factors on D4 conversion, MW of the polymer and dispersion average particle size observed in the studies conducted on the cationic emulsion polymerization of D4 in the presence of DBSA is summarised in Supplementary Table S1 in the Supplementary Materials. The mechanism of the cationic emulsion polymerization of D4 in the presence of DBSA was proposed [7] and the role of the induction period was explained based on detailed studies of intermediate products formed in this reaction [3].

**Figure 2:**
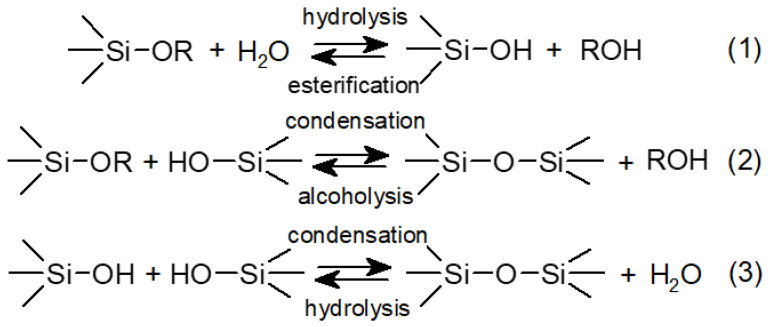
Process of synthesis of silicone polymer by hydrolysis of alkoxysilanes (**1**) and subsequent polycondensation ((**2**) and (**3**)). If tralkoxysilanes were used as starting materials in this reaction, a polymer with a crosslinked or even three-dimensional structure would be formed.

Polysiloxanes can also be obtained by the hydrolysis of alkoxysilanes and subsequent polycondensation involving silanol groups. This process is usually conducted in water in the presence of surfactants and is catalysed by acids or bases, so if DBSA was used as a surfactant, it could facilitate the process at the same time. The involved reactions are presented in Figure 2.

In the patent literature, there are many examples showing how that process can be implemented in practice [17,18,19,20,21]. Obviously, if a difunctional alkoxysilane is used, a linear polymer would be obtained, and if the alkoxysilane is trifunctional, the polymer would be partly crosslinked. The process of the synthesis of such partly crosslinked PDMS by the hydrolysis of methyltrimethoxysilane (METMS) in the presence of DBSA and non-ionic surfactants was studied in detail [10]. It was found that there was a maximum concentration of DBSA, at which the lowest particle size of the resulting silicone polymer dispersion could be obtained. Similar results were reported for the hydrolysis of METMS conducted using a base (NaOH) as the catalyst [22]. However, so far, no detailed study has been published that would deal with the process of obtaining a silicone polymer dispersion by co-reacting D4 and alkoxysilanes in an aqueous emulsion using DBSA and, possibly, also other surfactants, though such a process was patented already in 2001 [23]. Polysiloxane obtained in that patented process contained linear segments originating from polymerized D4 and branched segments originating from the hydrolysis of trialkoxysilanes. The synthesis of partly crosslinked silicone resin in an aqueous emulsion by polymerizing the mixtures of D4 with methacryloxypropyl trimethoxysilane (MATMS) and tetraethoxysilane (TEOS) was investigated [24,25,26] before and after that patent was granted, but the course of such a reaction has never been studied. The aqueous dispersions of silicone polymers obtained in these works were later used as starting dispersions for the emulsion polymerization of acrylic monomers. Recently, a comprehensive review of the synthesis, characterization and application of aqueous dispersions containing silicones was published [27].

In our study, we investigated the course of the process of the cationic polymerization of D4 in an aqueous emulsion using DBSA as the polymerization catalyst and surfactant in two different mixtures with trialkoxysilanes, so that the hydrolysis of trialkoxysilanes followed by polycondensation proceeded simultaneously. The course of the cationic emulsion polymerization of D4 alone was also studied for comparison. In our process, the monomers were added dropwise to the aqueous solution of DBSA (so called the “starved feed” mode). We selected this approach instead of a batch emulsion polymerization since it ensured a smoother run of the process and better repeatability needed for industrial implementation. It was also reported earlier that a lower particle size could be obtained if the process was carried out in such a “starved feed” mode [16]. The selected aqueous silicone polymer dispersions obtained in our process were used as starting dispersions for the synthesis of silicone–acrylic aqueous dispersions with a hybrid particle structure designed as the binder for architectural paints [28,29,30].

As it was proved in the previous studies, e.g., [31,32,33] FTIR can be an efficient tool that allows for the monitoring of the course of reactions involving silicone monomers. In our study, the results obtained from FTIR ATR were supported by the determination of the development of solids (i.e., polymeric reaction product) content as well as the toluene-insoluble fraction of the polymer and dispersion particle size in the course of the reaction and with gas chromatography (GC), as well as ^29^Si NMR investigations. The development of molecular weight was studied by gel permeation chromatography (GPC) only for the polymerization of D4 alone, since when D4 was polymerized in the mixtures with trialkoxysilanes, a partly crosslinked polymer was formed that was insoluble in solvents used in that technique.

## 2. Experimental Section

### 2.1. Starting Materials

Octamethylcyclotetrasiloxane (D4) (98%) was obtained from Momentive (Leverkusen, Germany). Methyltriethoxysilane (MTES) and vinyltriethoxysilane (VTES) (both technical grade) were obtained from Evonic (Warsaw, Poland) and 4-dodecylbenenzenesulfonic acid (DBSA) (96%) was obtained from PCC Exol (Brzeg Dolny, Poland). NaHCO_3_ (analytical grade) was obtained from ChemPUR (Plymouth, MI, USA). Demineralized water used in synthesis of aqueous silicone resin dispersions had conductivity of 0.06 μS. Methylene chloride for GC investigations and toluene for investigations of polymer solubility (both analytical grade) were obtained from Aldrich (Darmstadt, Germany).

### 2.2. Synthesis of Silicone Polymer Aqueous Dispersions

Synthesis of silicone polymer aqueous dispersions was conducted in 2 dm^3^ glass reactor equipped with disk-type stirrer, pump for dosing the monomers and reflux condenser that could be changed to distillation condenser. Three monomer compositions were investigated:

(1) D4 alone;
(2) Mixture of D4—84.0%; MTES—9.5%; VTES—6.5%;
(3) Mixture of D4—88.0%; VTES—12%.

The FTIR ATR sensor situated in the tip of the elastic probe connected to ReactIR 15^TM^ apparatus (see Section 2.3 below) was placed inside the reacting mixture. First, DBSA was dissolved in 875 g of demineralized water to obtain 0.5% solution, and then temperature was raised to 87–88 °C. After the desired temperature was reached, 234 g of monomers (either D4 or mixture of D4 with trialkoxysilanes) was pumped continuously to the reactor over 3.5 h. Next step was heating the reaction mixture at the same temperature for 1 h and, eventually, the reaction was completed by further heating the reaction mixture under reduced pressure of 0.2 Bar at the same temperature for 3 h with simultaneous distillation of alcohol/water azeotrope. Then, the reaction mixture was cooled to 25–30 °C and pH was fixed at 6.5 with NaHCO_3_ to obtain the final product—aqueous dispersion of silicone polymer.

**Figure 3:**
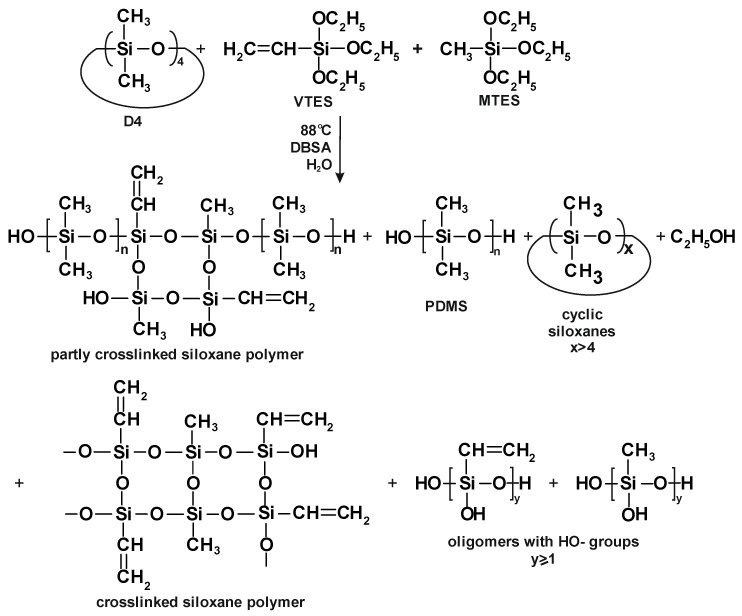
The process of cationic emulsion polymerization of D4 in the mixture with VTES and MTES.

The process of D4 polymerization in the mixture with trialkoxysilanes (MTES and VTES) is presented in Figure 3.

### 2.3. FTIR Studies of Cationic Emulsion Polymerization of D4 Alone and in Mixtures with Trialkoxysilanes

FTIR ATR spectra were recorded “in situ” (at a real time) during the reactions using ReactIR 15^TM^ apparatus supplied by METTLER TOLEDO—see Supplementary Figure S1 in Supplementary Materials for the image of the apparatus (a) and the idea of taking FTIR ATR spectra by the sensor placed in the probe tip (b). The elastic probe (FiberConduit^TM^) connected to the apparatus used fibres composed of AgX for transferring IR radiation from the source to the sensor that was in contact with the reaction mixture and then back to the mercury–cadmium–tellurium detector that was cooled with liquid nitrogen. The scans (256 scans per minute) were taken by the apparatus within the range 2800–700 cm^−1^. In order to confirm that the React IR could be an effective tool for in situ investigation of polymerization of silicone monomers in aqueous emulsion, a React IR probe was inserted first in D4 and then in emulsion of D4 in water, and it was found that the FTIR ATR spectra, thus, obtained were identical. This experiment confirmed that REACT IR 15^TM^ equipment could be effectively used in our process that proceeded in aqueous emulsion.

### 2.4. Determination of Solids Content of Samples Taken in the Course of Reaction

In total, ca. 0.2 g samples of the reaction mixture taken in the course of reaction after 30 min, 120 min, 210 min (end of addition of monomers), 270 min (end of final heating) and 450 min (end of vacuum distillation) were treated with 7.7% aqueous NaHCO_3_ solution to reach pH = 6.2–6.5, weighted and then dried in an oven for 1 h at 85 °C, followed by 4 h at 125 °C. Then, the samples were weighed again and the solids content (S) was calculated from Equation (1).

S = m_1_/m_0_ × 100% (1)

where:

m_0_—mass of the sample taken from the reactor;
m_1_—mass of the dried sample.

### 2.5. Determination of Average Particle Size and Particle Size Distribution of Samples Taken in the Course of Reaction

Particle size and particle size distribution of samples taken as described in Section 2.4 were conducted using light-scattering method (Malvern Zetasizer apparatus, Malvern Analytical Ltd., Malvern, UK).

### 2.6. Determination of Percentage of Toluene-Insoluble Fraction of Silicone Polymer Formed in the Course of Reaction

In total, ca. 1 g samples were taken from the reaction mixture, dried at room temperature for 20 hrs and then at 65 °C until no weight change was observed. Then, portions of dry solids (ca. 0.1 g) were weighed and placed in 10 cm^3^ of toluene contained in closed glass cups and left for 20 h at 23 °C. After that time, the samples were taken from toluene, dried at 65 °C until no weight change was noticed and the percentage of toluene-insoluble fraction was calculated from the equation:

% of toluene-insoluble fraction = m_1_/m_0_ × 100% (2)

where:

m_0_ = mass of the sample before test;
m_1_ = mass of the sample after complete drying.

### 2.7. GC Analysis

Test samples (ca. 0.5 g) were prepared by adding 2 cm^3^ of dichloromethane and TEOS as an internal standard to samples taken from the reactor in the course of the process. Standard stock solution was prepared by weighing 300 mg of each analyte into a volumetric flask containing some dichloromethane; series of dilutions were performed. The extracted solution was injected into a GC system. The analyses were performed in SHIMADZU GC-2010 gas chromatography system equipped with a flame ionization detector (FID). The capillary column DB-1(60 m × 0.25 mm × 1 µm, 100% PDMS, Agilent J&W, Agilent, Santa Clara, CA, USA) was used. The optimized GC temperatures were 250 °C at the injector port and 310 °C at the detector. The GC oven was held at 80 °C for 10 min and then ramped at 10°C/min to 200 °C, held for 6 min and then ramped to 300 °C at 40 °C/min and held for 4.5 min.

### 2.8. GPC Investigations

GPC investigations were carried out at 80 °C on SHIMADZU chromatograph using toluene as eluent and poly(methyl methacrylate) reference samples (Polymer Standards Service) for column calibration. The column was Phenogel 5 µ Linear (300 × 7.8 mm), Phenomenex, Torrance, CA, USA and the low rate was 1 cm^3^/min.

### 2.9. ^29^Si NMR Studies

Studies of the reaction product by solid state ^29^Si NMR were conducted on AVANCE III 400 spectrometer (BRUKER, Billerica, MA, USA) working with resonance frequency of 79.495 MHz; ^29^Si NMR spectra were registered using One Pulse (High Power Decoupling Magic Angle Spinning) technique.

## 3. Results and Discussion

The course of the processes of the synthesis of polysiloxanes can be effectively studied by FTIR, primarily based on the changes of intensity and position of bands corresponding to stretching vibrations of the Si–O bond. In general, it is known that the bands in 1026–1020 cm^−1^ range correspond to Si–O in long polysiloxane chains, while the bands in 1083–1069 cm^−1^ range can be attributed to Si–O in cyclic compounds (such as D4, D5 or higher cycles) and in the crosslinked or branched structures. The “cage” structures present in silsesquioxanes can usually be identified by an inflection at 1140 cm^−1^ [6]. In more complex systems, these bands may overlap, so it is difficult to observe how they change when polymerization is progressing. REACT IR 15^TM^ equipment used in our study allowed for searching the changes in these bands in the course of polymerization in a real time, and based on these data supported by GC, the development of the toluene-insoluble fraction of the polymer and gravimetric investigations, we could learn when the reaction started and how fast it proceeded.

In order to follow these changes, the FTIR ATR spectra of monomers used in the investigated processes were first created as described in Section 2.3, and the most specific bands were identified based on [31,34,35,36]—see Supplementary Figures S2–S4 and Supplementary Tables S2–S4 in the Supplementary Materials.

### 3.1. Studying the Course of Cationic Emulsion Polymerization of D4 Alone and in the Mixtures with Alkoxysilanes

#### 3.1.1. FTIR Studies

**Figure 4:**
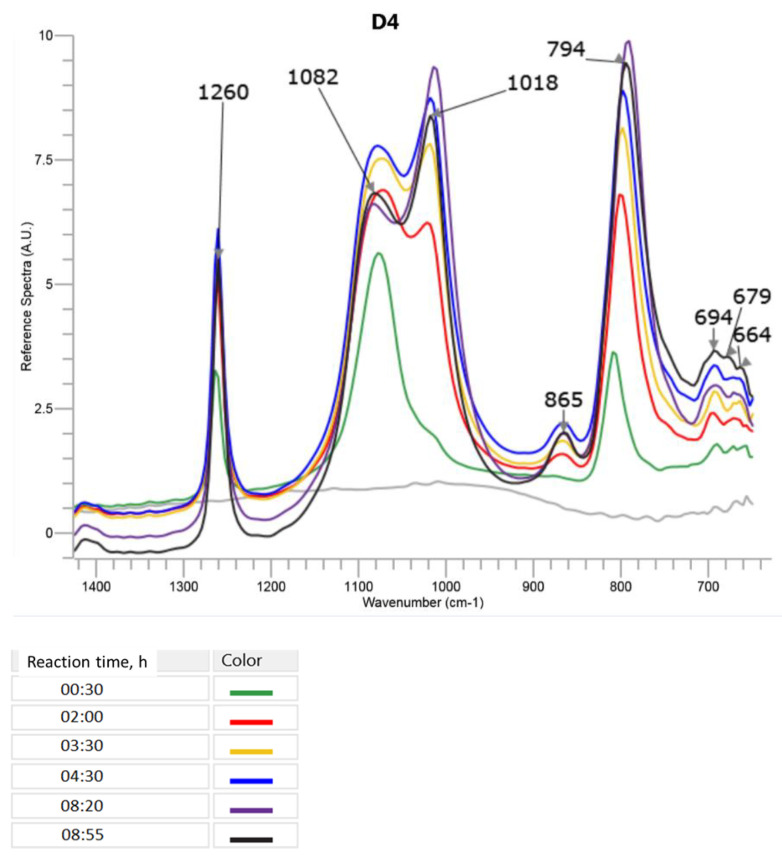
FTIR ATR spectra recorded in the course of cationic emulsion polymerization of D4 alone. Only the range 1400–700 cm^−1^, where the significant changes were observed, is shown. The colours of lines corresponding to the spectra recorded at increasing reaction times are displayed.

The changes in the most specific bands in the FTIR ATR spectrum during the cationic emulsion polymerization of D4 alone are presented in Figure 4 (only a part of the spectrum between 1400 cm^−1^ and 700 cm^−1^, where the most significant changes were observed, is shown). A three-dimensional picture of the spectra taken by the ReactIR 15TM apparatus in the course of the reaction can be found in Supplementary Figure S5 in the Supplementary Materials.

It can be observed in Figure 4 that already at the beginning of the process (30 min after the addition of D4 started), the band with the maximum at 1053 cm^−1^ corresponding to Si–O–Si in D4 disappeared, confirming that the opening of the D4 ring occurred very fast in the presence of DBSA. At the same time, in the course of the reaction, two new peaks appeared with maxima at 1082 cm^−1^ and 1018 cm^−1^ that, in this case, could be attributed to Si–O–Si in bigger cyclic and linear polysiloxane structures, respectively. It can be noticed from Figure 4 how the intensity of the band corresponding to the linear structures increased in the course of the reaction and, at the same time, the intensity of the band corresponding to the cyclic structures decreased. The band at 865 cm^−1^ that was not present in the spectrum of D4 alone and increased in the course of the reaction could be attributed to Si–OH [34].

**Figure 5:**
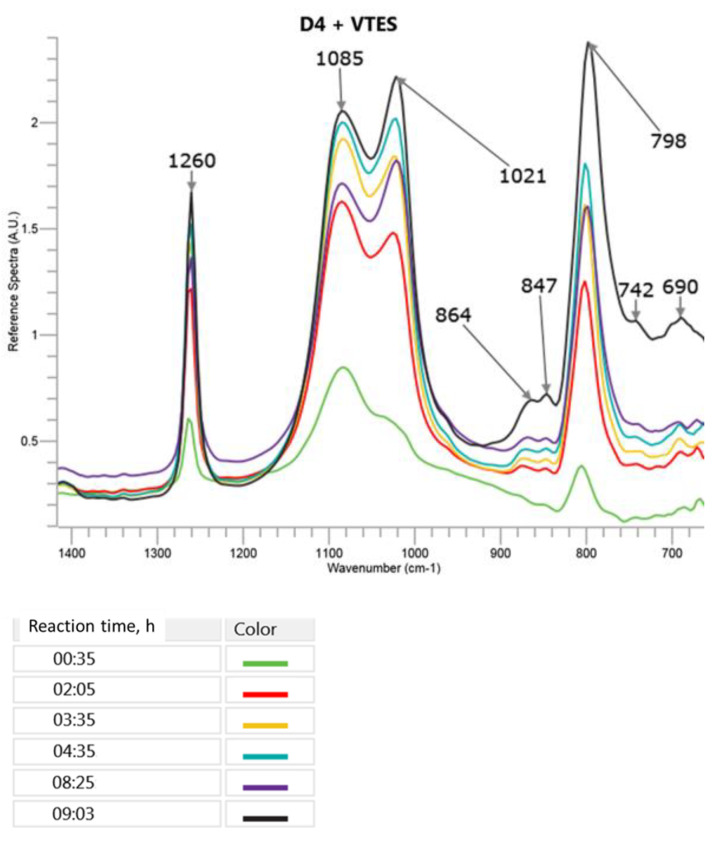
FTIR ATR spectra recorded in the course of cationic emulsion polymerization of D4 in the mixture with VTES. Only the range 1400–700 cm^−1^, where the significant changes were observed, is shown. Tthe colours of lines corresponding to the spectra recorded at increasing reaction times are displayed.

The changes in the most specific bands in the FTIR ATR spectra during the cationic polymerization of D4 in the mixture with VTES are presented in Figure 5 (only a part of the spectrum between 1400 cm^−1^ and 700 cm^−1^, where the most significant changes, was observed). A three-dimensional picture of the spectra taken by the React IR 15^TM^ apparatus in the course of the reaction can be found in Supplementary Figure S6 in the Supplementary Materials.

It can be observed in Figure 5 that the presence of a small amount of VTES did not result in a significant change of the FTIR ATR spectra taken in the course of the reaction as compared to the polymerization of D4 alone. Only the ratio of the intensity of the new 1085 cm^−1^ band to the new 1021 cm^−1^ band was higher than in the case of the 1082 cm^−1^ and 1018 cm^−1^ bands appearing in the polymerization of D4 alone. This phenomenon could be explained by the formation of branched and partly crosslinked structures due to the process of the hydrolysis of VTES and the subsequent polycondensation that was not possible during the polymerization of D4 alone. The bands at 864 cm^−1^ and 847 cm^−1^ that were not present in D4 and VTES spectra and increased in the course of the reaction could be attributed to Si–OH in -Si(CH_3_)_2_–OH and in -Si (O) (CH=CH_2_)–OH, respectively.

**Figure 6:**
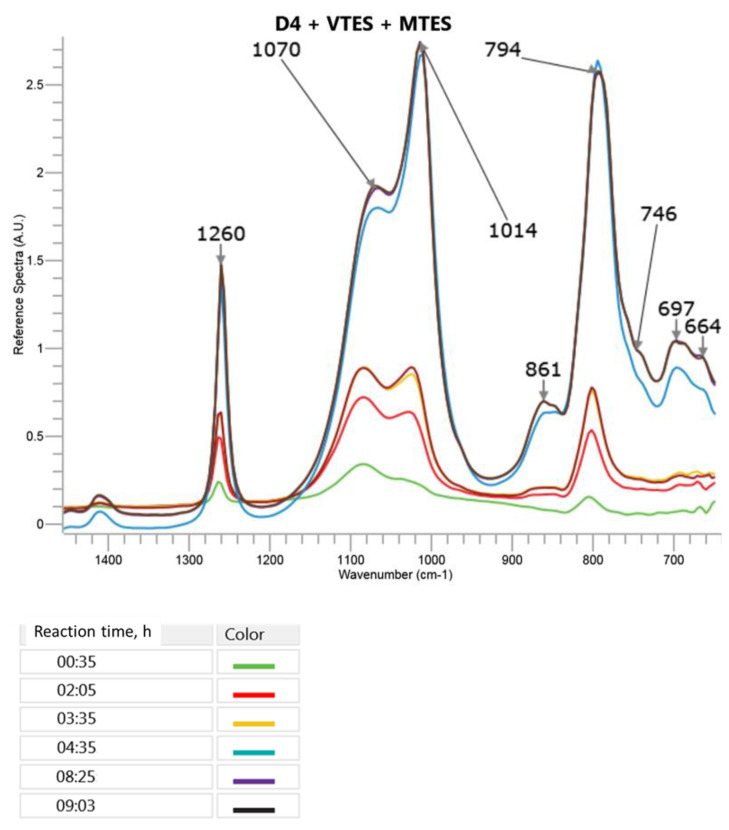
FTIR ATR spectra recorded in the course of cationic emulsion polymerization of D4 in the mixture with MTES and VTES. Only the range 1400–700 cm^−1^, where the significant changes were observed, is shown. Tthe colours of lines corresponding to the spectra recorded at increasing reaction times are displayed below.

The changes in the most specific bands in the FTIR ATR spectra during the cationic emulsion polymerization of D4 in the mixture with MTES and VTES are presented in Figure 6 (a part of the spectrum between 1400 cm^−1^ and 700 cm^−1^, where the most significant changes were observed). A three-dimensional picture of the spectra taken by the ReactIR 15TM apparatus in the course of reaction can be found in in Supplementary Figure S7 in the Supplementary Materials.

It can be observed in Figure 6 that in the course of the reaction, the intensity of the band corresponding to the cyclic and branched or crosslinked structures (1070 cm^−1^) increased slower than in the case of the polymerization of D4 in the presence of VTES only. Moreover, the proportion of the intensities of that band and the band corresponding to the linear structures was different than the one observed in the process, where only VTES was used in the mixture with D4. The reason could be a higher share of trialkoxysilanes in the monomer mixture (16%) as compared to the process where the mixture of D4 with VTES only was applied (12%). The broad band at 861 cm^−1^ that was not present in the D4, VTES and MTES spectra and increased in the course of the reaction could be attributed to the overlapped signals of Si–OH in -Si(CH_3_)_2_–OH, -Si(O)(CH_3_)–OH and -Si(O)(CH=CH_2_)–OH.

In order to verify the observations determined based on the FTIR studies presented in Section 3.1.1 above, the progress of the silicone polymer formation (the development of the solids content and toluene-insoluble fraction) as well as the changes in the dispersion particle size and silicone monomers content in the course of the polymerization process were also investigated—see Section 3.1.2, Section 3.1.3, Section 3.1.5 and Section 3.1.6, respectively.

#### 3.1.2. Solids Content Development in the Course of Reaction

**Figure 7:**
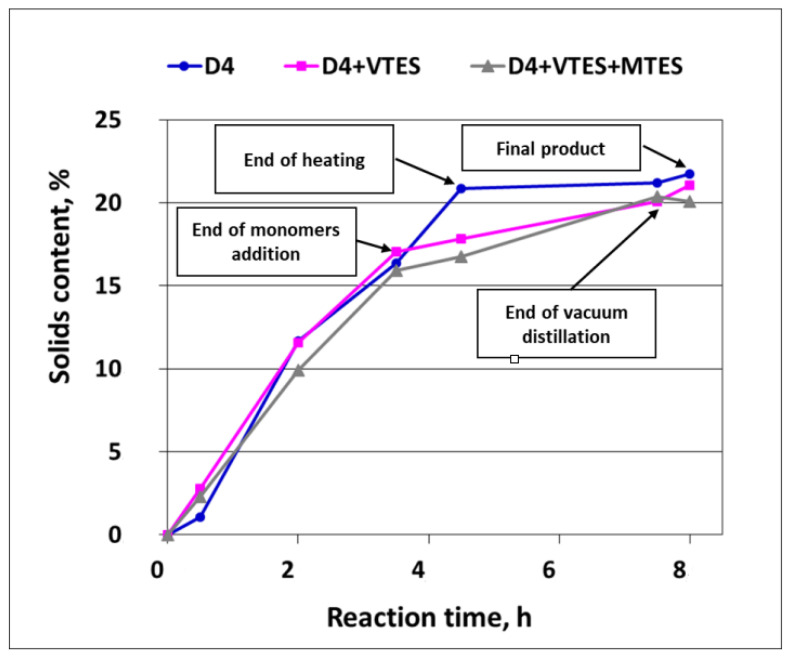
Development of solids content (polymer mass) in the course of cationic emulsion polymerization of D4 alone and in the mixtures with alkoxysilanes.

The development of the solids content (polymer mass) in the course of the polymerization of D4 alone and in the mixtures with alkoxysilanes is shown in Figure 7.

It is clear from Figure 7 that the development of the solids content (i.e., the development of the silicone polymer formation) in the first 0.5 h of the process was retarded, especially in the case of the polymerization of D4 alone. This observation confirmed the conclusions from the earlier studies [3], where it was proved that there was an induction period of ca. half an hour in the cationic emulsion polymerization of D4. It is also clear from Figure 7 that the process was faster in that period when D4 was added in the mixtures with alkoxysilanes. The explanation of this phenomenon could be the immediate formation of reactive Si–OH groups due to the very fast hydrolysis of alkoxy groups of VTES and MTES in a very strongly acidic medium and at a quite high temperature (88 °C). Then, the reaction of the silicone polymer formation proceeded at a similar rate for D4 and for D4 in a mixture with alkoxysilanes, but only until the monomer addition was completed. As heating of the reaction mixture was continued, the process of the polymerization of D4 alone proceeded further with a similar rate, while the process of the polymerization of D4 in the mixture with alkoxysilanes slowed down. The reason for that could be a steric hindrance resulting from branched or even partly crosslinked polymer structures that were formed in the presence of trifunctional reactants (MTES and VTES) along with D4 and the lack of a supply of low MW monomers, which could react faster.

#### 3.1.3. Development of Toluene-Insoluble Fraction of Silicone Polymer in the Course of Reaction

**Figure 8:**
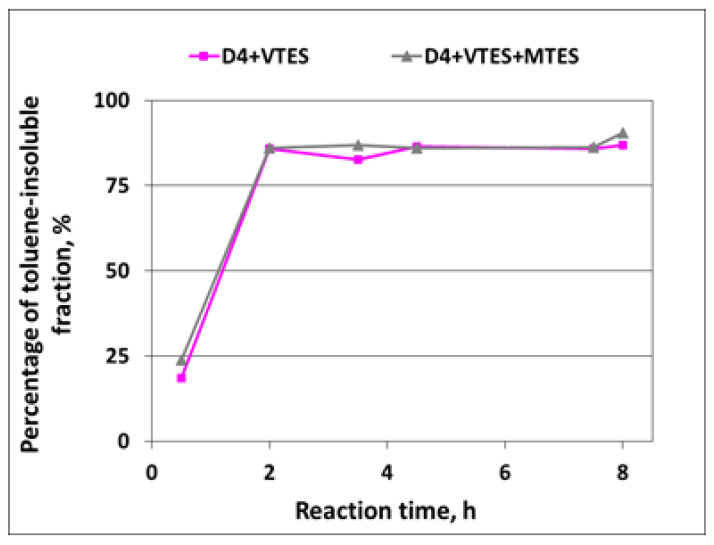
Development of toluene-insoluble fraction of silicone polymer in the course of cationic emulsion polymerization of D4 in the mixtures with alkoxysilanes.

In the polymerization of D4 alone, no toluene-insoluble fraction was detected—all samples taken in the course of reaction were soluble in toluene when tested according to the method described in Section 2.6, so it was proved that a linear silicone polymer was formed. The development of the toluene-insoluble fraction of the silicone polymer in the course of the polymerization of D4 in the mixtures with alkoxysilanes is shown in Figure 8.

From the results shown in Figure 8, it is clear that partly crosslinked or crosslinked polymer structure was formed very quickly—already after 2 h, the percentage of the toluene-insoluble fraction exceeded 80%. It is also clear that D4 must have reacted with alkoxysilanes producing a partly crosslinked silicone copolymer, since, if the D4 homopolymer and product of the polymerization of alkoxysilanes were formed separately, the percentage of the toluene-insoluble fraction would be much lower due to the very low proportion of alkoxysilanes to D4 (12/88 in the case of the reaction of D4 in the mixture with VTES and 16/84 in the case of the reaction of D4 in the mixture with VTES + MTES).

#### 3.1.4. Development of Silicone Polymer Average Molecular Weight in the Course of Reaction

**Figure 9:**
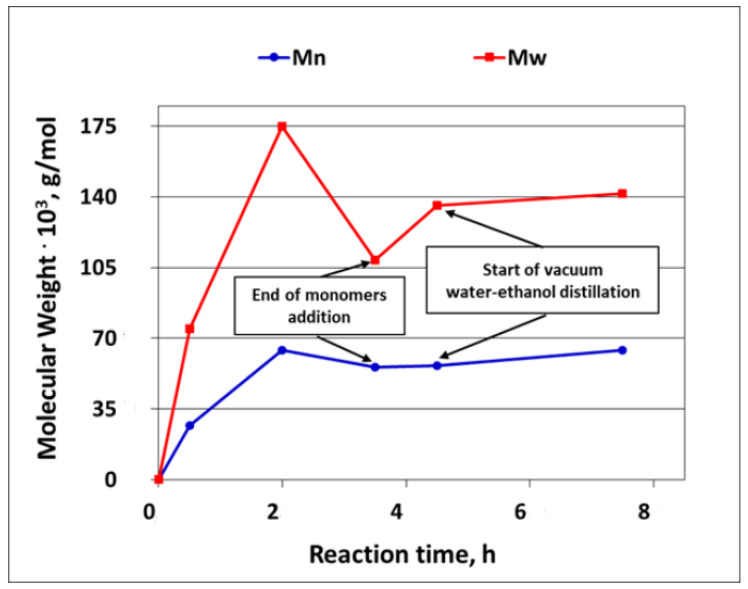
Development of Mw and Mn of silicone polymer (PDMS) in the course of cationic emulsion polymerization of D4 alone.

As it was explained earlier in this paper, the development of the silicone polymer average molecular weight in the course of the reaction could only be studied for the polymerization of D4 alone, since, when D4 was polymerized in the mixtures with trialkoxysilanes, the reaction product was only partly soluble in solvents applied in GPC determinations. The development of the Mw and Mn of the silicone polymer (PDMS) in the course of D4 polymerization is shown in Figure 9.

From the results presented in Figure 9, it is clear that the average molecular weight of the polymer increased fast in the first 2 h and then dropped, most probably because at this stage, the reverse reaction started to prevail (see Figure 1). The further heating of the reaction mixture for 1 h without the addition of a monomer resulted in only a slight increase in the Mw and Mn that continued until the end of the process. It was also found that the oligomeric fraction with the Mw of ca. 900 was formed already in the beginning of the process and remained in the reaction product—see Supplementary Figure S8 in the Supplementary Materials, where the full GPC chromatogram of the reaction product is presented. This GPC chromatogram of the reaction product was very similar to the one obtained in the studies on the cationic emulsion polymerization of 1,3,5,7-tetramethylcyclotetrasiloxane (^H^D4), where the formation of a low molecular weight fraction was also confirmed [37].

#### 3.1.5. Particle Size Development in the Course of Reaction

**Figure 10:**
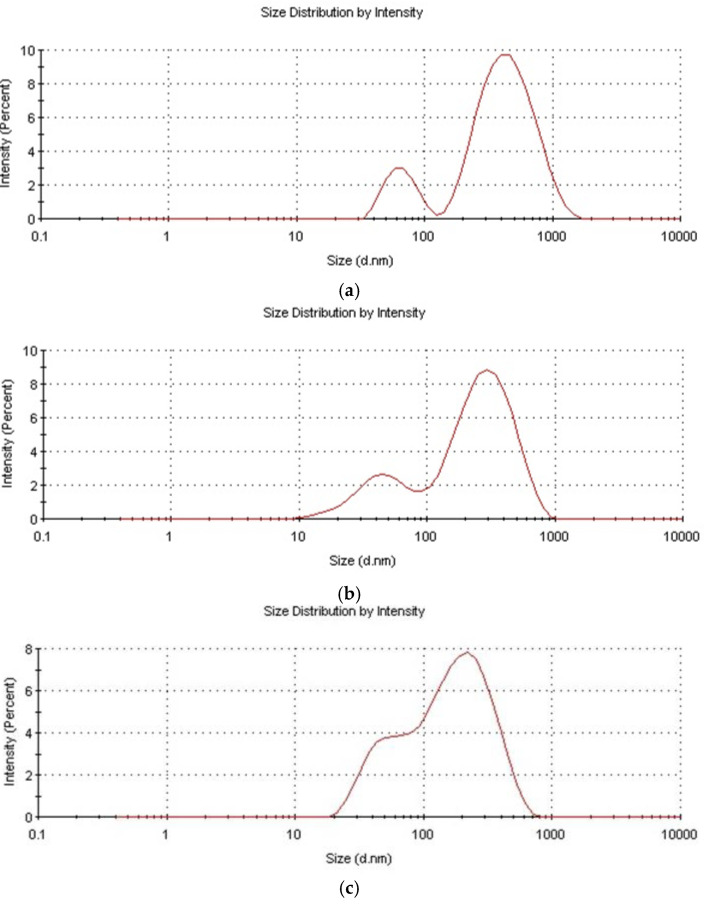
Particle size distribution of aqueous silicone polymer dispersions obtained in the study. (**a**)—D4; (**b**)—D4 + VTES; (**c**)—D4 + VTES + MTES.

The particle size distribution was bimodal for all samples, though a fraction of low size particles was relatively small—see Figure 10. The reason for the bimodal particle size distribution could be the starved feed mode of the process, which facilitated the secondary nucleation. A similar phenomenon was also observed in the process of the starved feed emulsion polymerization of acrylic monomers [38]. The reason for the generally smaller particle size when D4 was polymerized in the mixtures with alkoxysilanes could be the lower hydrophobicity of particles resulting from the presence of much more Si–OH groups on the particle’s surface. The polydispersity index was 0.15–0.5 for all samples, and generally increased in the course of the reaction reaching, a ca. 0.5 value for the final products. The broadening of the particle size in the course of the reaction was also observed earlier in the study in the emulsion polymerization of D4, and was explained by the increased probability of the reactions among the polymerization reaction sites [14].

#### 3.1.6. GC Investigations

**Figure 11:**
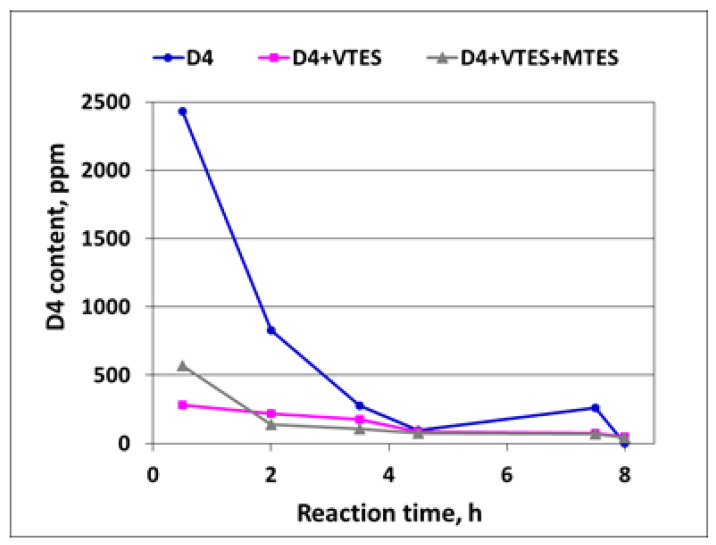
Decrease in D4 content in the course of cationic emulsion polymerization of D4 alone and in the mixtures with alkoxysilanes as determined by GC.

A decrease in the D4 content in the course of the polymerization of D4 alone and in the mixtures with alkoxysilanes as determined by GC is shown in Figure 11.

It is clear from Figure 11 that after the induction period, the D4 content was much higher in the case of the polymerization of D4 alone than in the case of the polymerization of D4 in the mixtures with alkoxysilanes, proving again that the reaction in that period proceeded much faster in the latter case. Then, the D4 content started to decrease very fast and was not detected in the final product.

The D3, D5 and alkoxysilanes content was also determined by GC. No unreacted alkoxysilanes were detected in the final product and D3 was absent in all samples. The absence of D3 could be expected taking into account that it is a strained cycle due to its planar structure. D5 was detected only in samples taken in the course of the polymerization of D4 alone. Changes in the D5 content in that reaction are presented in Supplementary Figure S9 in the Supplementary Materials.

We found that, during the addition of D4, the content of D5 first increased and then started to decrease to reach a stable level of ca. 100 ppm—see Supplementary Figure S9 in the Supplementary Materials. Such an increase in the D5 content in the initial stage of D4 polymerization and decrease in the next stages was also observed earlier [11].

D3 (hexamethyloctatrisiloxane), D5 and alkoxysilanes were not detected in samples taken in the course of the polymerization of D4 in the mixtures with alkoxysilanes. The lack of D3 in all samples taken in the course of the polymerization of D4 alone confirmed the conclusion from the studies on the mechanism of the cationic emulsion polymerization of D4 alone, that D3 may only be formed as an intermediate and not as a side product [3]. The formation of D5 in the course of the polymerization of D4 alone was also confirmed [3,11]. The observed lack of alkoxysilanes in samples taken in the course of the polymerization of D4 in the mixtures with alkoxysilanes became obvious when taking into account that in the severe reaction conditions, the hydrolysis of alkoxysilanes proceeded rapidly—see also Section 3.1.2.

**Figure 12:**
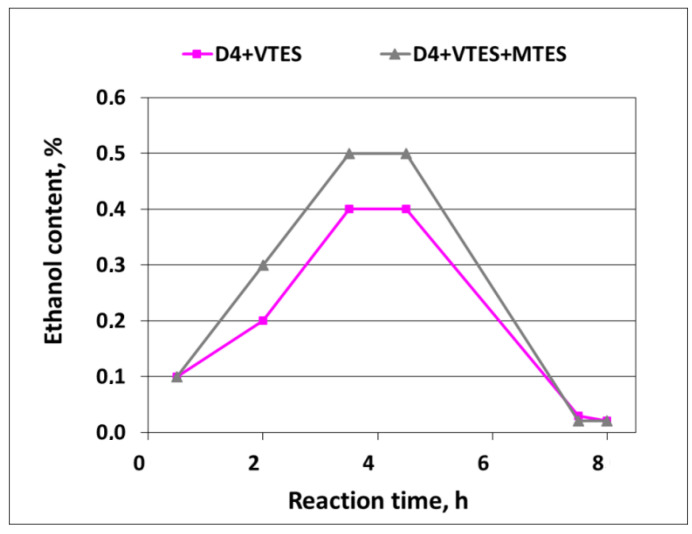
Ethanol formation in the course of cationic emulsion polymerization of D4 in the mixtures with alkoxysilanes.

Ethanol formation in the course of the polymerization was also confirmed by GC—see Figure 12.

As it can be noticed from Figure 12, the formation of ethanol in the course of the reaction (due to the hydrolysis of alkoxysilanes) already started when the process started, and then proceeded smoothly to reach the final content of ca. 0.4–0.5% at the end of the addition of D4 in the mixtures with alkoxysilanes. Finally, it dropped to trace amounts after the vacuum distillation of the ethanol–water azeotrope was completed. The amounts of ethanol that were actually detected in the distillate constituted 89% of the theoretical amount in the case of the mixture of D4 with VTES and 85% of the theoretical amount in the case of the mixture of D4 with VTES and MTES. Since alkoxysilanes were not detected by GC in the samples of the final reaction products, it could be anticipated that hydrolysis was complete and the differences between the actual and theoretical amounts of ethanol resulted, apparently, from the losses during vacuum distillation.

#### 3.1.7. ^29^Si NMR Investigations

**Figure 13:**
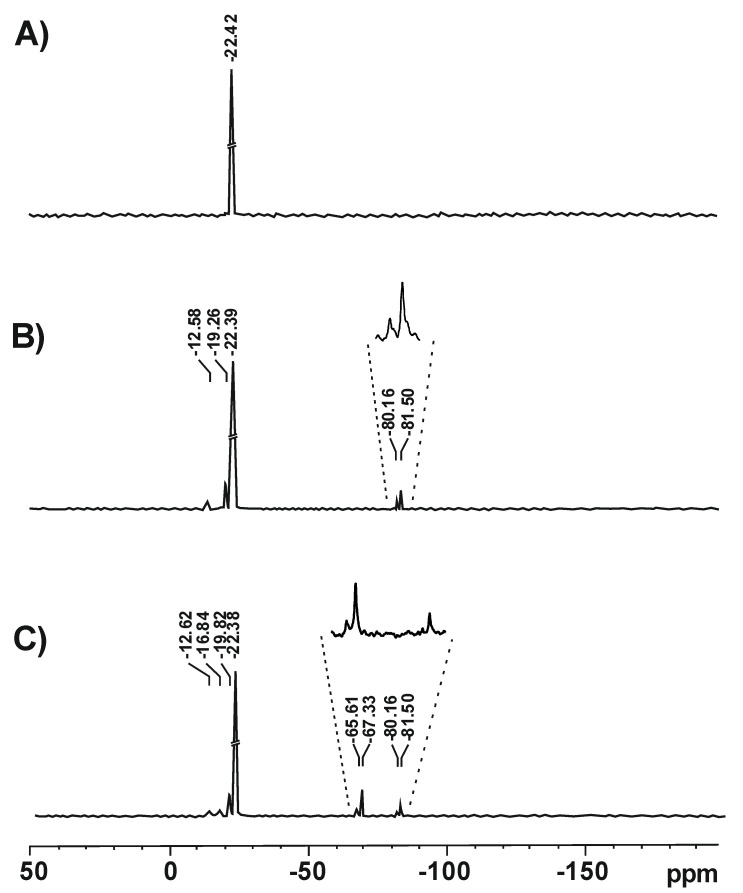
The ^29^SiNMR spectra of the reaction products obtained in cationic emulsion polymerization of D4 alone (**A**), D4 in the mixture with VTES (**B**) and D4 in the mixture with VTES and MTES (**C**).

The structure of silicone polymers obtained in the polymerization of D4 alone and in the mixtures with alkoxysilanes was confirmed by solid-state ^29^Si NMR investigations. The relevant spectra are shown in Figure 13.

In the spectrum of the polymer obtained in the polymerization of D4 alone, only one signal at −22.42 ppm was observed that corresponded to the linear -O–Si(CH_3_)_2_–O- structure [37]. In the spectra of polymers obtained in the polymerization of D4 in the mixtures with VTES and VTES + MTES, there were signals corresponding to the linear structure (−22.39 ppm/−22.38 ppm), but also much less intense additional signals were observed that corresponded to oligomeric structures with silanol groups, e.g., (CH_2_=CH(OH)_2_Si)_2_O (−12.58 ppm/−12.62 ppm) or CH_3_(OH)_2_Si)_2_O (−16.84 ppm) [38], cyclic (-O–Si(CH_3_)_2_–O-)_x_ structures (−19.26 ppm/−19.82 ppm) [39] as well as branched or crosslinked structures CH_2_=CH–Si–(O–Si)_3_ (−81.50 ppm/−81.50 ppm) [40] and CH_3_–Si–(O–Si)_3_ (−65.61 ppm/67.53 ppm) [39]. It should be noted that no signal from the VTES monomer at −58.26 ppm [41] was found, proving that akoxysilanes fully hydrolysed in the course of the reaction.

## 4. Conclusions

The results of a “real-time” investigation of the reaction of the cationic emulsion polymerization of D4 in the mixtures with alkoxysilanes by the FTIR ATR, GC, development of the toluene-insoluble fraction of the polymer and gravimetric analysis proved that the silicone polymer formation proceeded faster than when D4 alone was polymerized, especially in the beginning of the process. The reason for that could be the immediate hydrolysis of alkoxy groups in a strongly acidic environment, leading to the formation of reactive silanol (Si–OH) groups. Though the process was conducted in a “starved feed” mode and at relatively high temperature (87–88 °C), the induction period of ca. 30 min reported earlier [3] was also observed. This was due to the fact that the hydrolysis of alkoxysilanes followed by polycondensation started immediately (see Figure 12), showing ethanol formation in the course of the reaction, but the ring opening of D4 followed by polymerization was somehow delayed (see Figure 7 showing an increase in polymer mass in the polymerization of D4 alone vs. in the polymerization of D4 in the mixtures with alkoxysilanes). The determination of the toluene-insoluble fraction content in the reaction product (see Figure 8) confirmed that mainly a partly crosslinked copolymer containing both linear -Si(R)_2_–O- moieties and branched R–Si(O_3_)- moieties was obtained in the process we studied (see simplified structures of the reaction products in Figure 3). The presence of these structures in the reaction product was confirmed by ^29^Si NMR. Thanks to the “starved feed” mode of conducting the process, the concentration of cyclic siloxanes (D4 and D5) remained at a very low level in the course of the reaction, and only traces were detected in the final product. The particle size development in the course of the reaction was also studied, and it was found that the particle size distribution was bimodal and was broadening as the reaction proceeded. However, this phenomenon was less distinct when D4 was polymerized in the mixtures with alkoxysilanes.

## Supplementary Materials

The following Supplementary Materials can be downloaded. Figure S1: REACT IR 15TM apparatus (a); Scheme that explains how FTIR ATR spectra are taken by the sensor placed in the probe tip (b); Figure S2: FTIR ATR spectrum of D4 recorded by ReactIR 15TM apparatus; Figure S3: FTIR ATR spectrum of VTES recorded by ReactIR 15TM apparatus; Figure S4: FTIR ATR spectrum of MTES recorded by ReactIR 15TM apparatus; Figure S5: FTIR ATR spectra recorded in the course of cationic emulsion polymerization of D4 alone—A three dimensional picture. Only the range 1400–700 cm^−1^ where the significant changes were observed is shown; Figure S6: FTIR ATR spectra recorded in the course of cationic emulsion polymerization of D4 in the mixture with VTES—Athree dimensional picture. Only the range 1400–700 cm^−1^ where the significant changes were observed is shown; Figure S7: FTIR ATR spectra recorded in the course of cationic emulsion polymerization of D4 in the mixture with VTES and MTES—A three dimensional picture. Only the range 1400–700 cm^−1^ where the significant changes were observed is shown; Figure S8: Full GPC chromatogram of the reaction product obtained in cationic emulsion polymerization of D4 alone; Figure S9: Changes in D5 content in the course of cationic emulsion polymerization of D4 alone as determined by GC; Table S1: Effect of various factors on D4 conversion, MW of the polymer and dispersion average particle size observed in the studies on cationic emulsion polymerization of D4 in the presence of DBSA; Table S2: Identification of the most specific bands in FTIR ATR spectrum of D4 shown in Figure S2; Table S3: Identification of the most specific bands in FTIR ATR spectrum shown in Figure S3; Table S4: Identification of the most specific bands in FTIR ATR spectrum shown in Figure S4.

## Sample Availability

Not available.
