# Supplementary material for: Cationic Emulsion Polymerization of Octamethylcyclotetrasiloxane (D4) in Mixtures with Alkoxysilanes
Source: Molecules. 2022 Jan 18;27(3):605. doi: 10.3390/molecules27030605 (PMC8839893; doi:10.3390/molecules27030605)
Supplement: Supplementary file 1 [file molecules-27-00605-s001.zip › molecules-1517714-supplementary.pdf]

Cationic emulsion polymerization of octamethylcyclotetrasiloxane (D4) in the mixtures with the alkoxysilanes

Janusz Kozakiewicz, Joanna Trzaskowska, Michal Kędzierski, Joanna Sołtysiak, Elżbieta U. Stolarczyk, Izabela Ofat-Kawalec, Jarosław Przybylski

Supplementary Materials

This "Supplementary Materials" file contains the following additional results of investigations on cationic emulsion polymerization of octamethylcyclotetrasiloxane (D4) alone and in the mixtures with vinyltrietoxysilane (VTES) and VTES + methyltriethoxysilane (MTES) :

Table S1 - Effect of various factors on D4 conversion, MW of the polymer and dispersion average particle size observed in the studies on cationic emulsion polymerization of D4 in the presence of DBSA.

Figure S1 - REACT IR 15TM apparatus (a); Scheme that explains how FTIR ATR spectra are taken by the sensor placed in the probe tip (b).

FiguresS2-S4– FTIR ATR spectra of silicone monomers (D4, VTES and MTES)

Tables S2-S4 – Identification of bands on FTIR ATR spectra of silicone monomers (D4, VTES and MTES)

Figures S5-S7 – Three dimensional pictures of FTIR ATR spectra taken in the course of cationic emulsion polymerization of D4, D4 + VTES and D4 + VTES + MTES

Figure S8 – Full chromatogram of the reaction product of cationic emulsion polymerization of D4 as obtained from Gel Permeation Chromatography (GPC). Figure S9 - Changes in D5 content in the course of cationic emulsion polymerization of D4 alone as determined by GC.

Table S1. Effect of various factors on D4 conversion, MW of the polymer and dispersion average particle size observed in the studies on cationic emulsion polymerization of D4 in the presence of DBSA.

	Observation	Reference
D4 conversion	Increased when DBSA concentration decreased and temperature increased.	[10]
	Increased when DBSA concentration decreased, but only until certain DBSA concentration (0.27%).	[11]
	Increased when DBSA concentration decreased, but only until certain DBS concentration (ca. 0.6%).	[12]
	Increased when DBSA concentration decreased and temperature increased and with the use of other surfactants along with DBSA. Was affected also by concentration and rate of addition of D4 and by stirring rate.	[13]
MW of the polymer	Increased when the reaction temperature decreased	[6, 10]
	Achieved a maximum at DBSA concentration equal to 2%.	[14]
Dispersion average particle size	Increased when DBSA concentration decreased.	[11, 14, 15]
	Was affected by the presence of co-surfactants.	[13]
	Was affected by the way D4 was introduced in the reaction mixture	[14, 16]

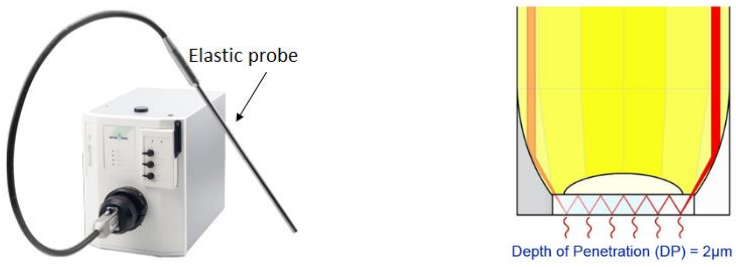

(a) **(b)**

Figure S1. REACT IR 15TM apparatus (a); Scheme that explains how FTIR ATR spectra are taken by the sensor placed in the probe tip (b).

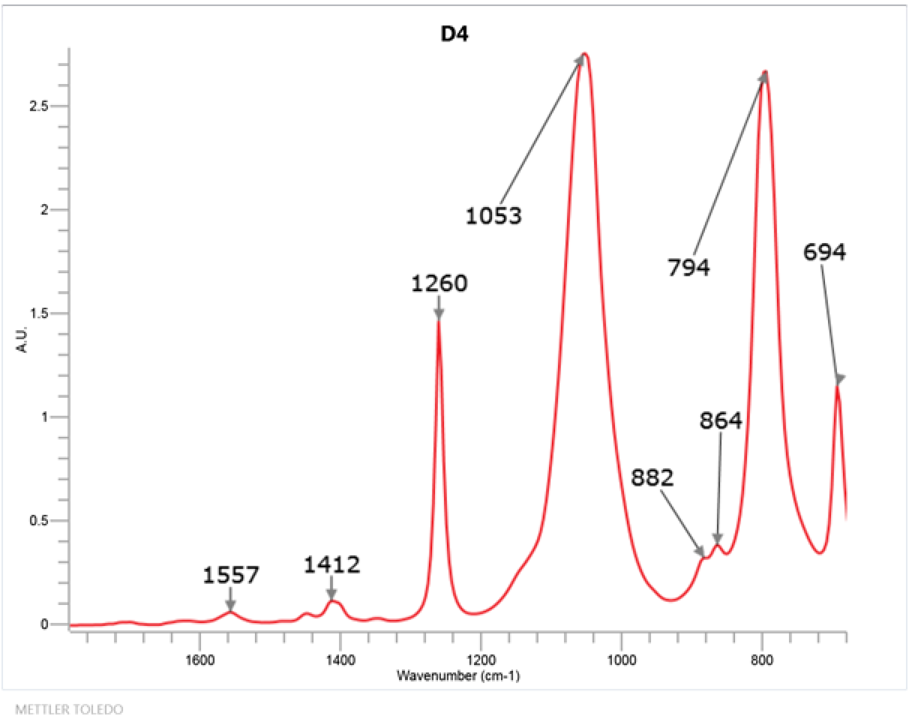

FigureS2. FTIR ATR spectrum of D4 recorded by ReactIR 15™ apparatus.

Table S2. Identification of the most specific bands in FTIR ATR spectrum of D4 shown in Fig. S2.

**Wavenumber, cm^{-1}**	**Identification**
1 260	H_3**C-Si**(O) symmetric bending
1 053	**Si-O**-Si stretching
864	**Si-(CH_3)$_2$** stretching
794	H_3**C-Si**(O) stretching

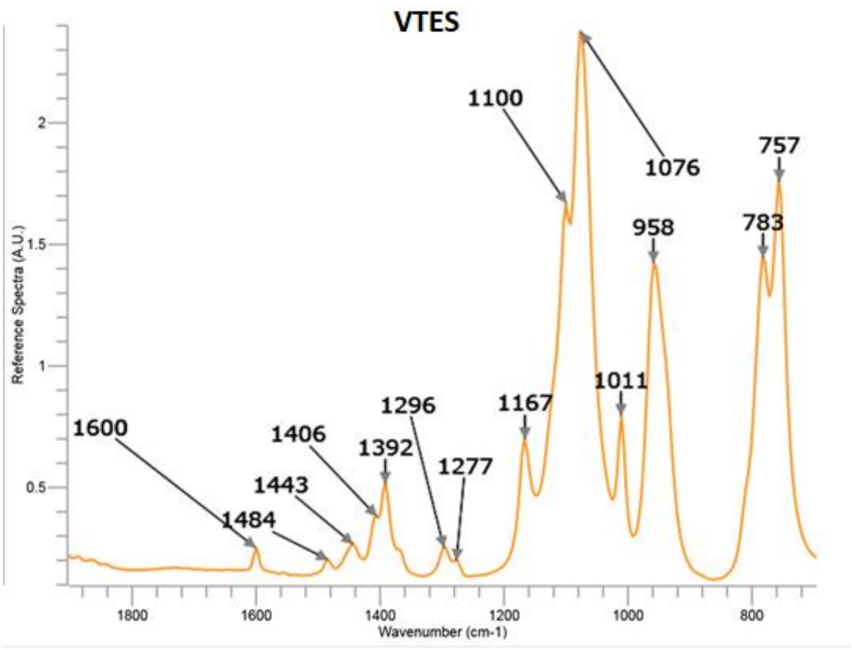

Figure S3. FTIR ATR spectrum of VTES recorded by ReactIR 15™ apparatus.

Table S3. Identification of the most specific bands in FTIR ATR spectrum shown in Fig. S3.

**Wavenumber, cm^{-1}**	**Identification**
1 400	==CH_2 scissors
1 277	H_3**C-Si**(O) symmetric deformation
1 167	CH_3 rocking
1 100, 1075	**Si-O**-C_2H_5 asymmetric stretching
1 011	C==C twist
958	**Si-O**-H stretching and ===CH_2 wagging
783, 757	H_3**C-Si**(O) symmetric stretching

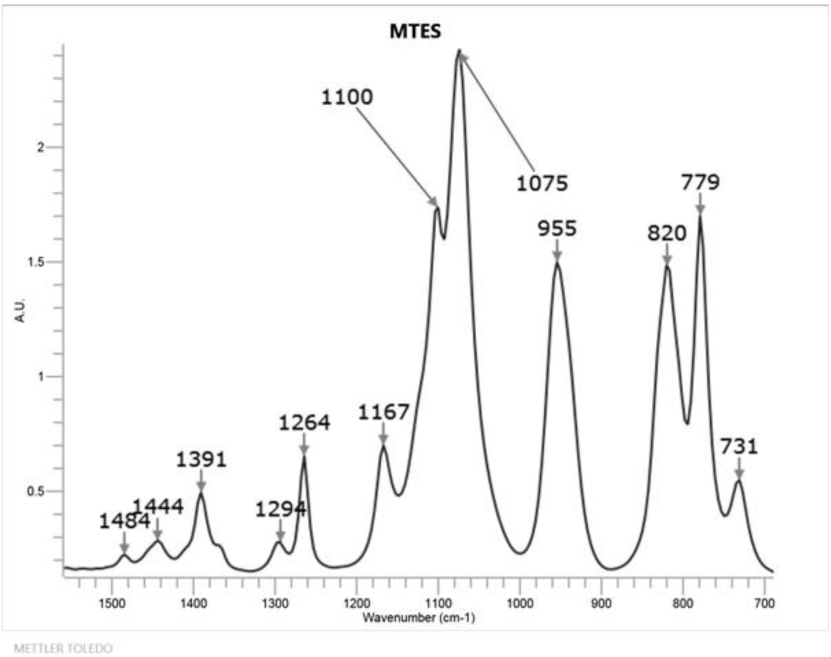

Figure S4. FTIR ATR spectrum of MTES recorded by ReactIR 15™ apparatus.

Table S4. Identification of the most specific bands in FTIR ATR spectrum shown in Fig.S43.

**Wavenumber, cm^{-1}**	**Identification**
1 264	H_3**C-Si**(O) symmetric deformation
1 167	CH_3 rocking
1 100, 1 075	**Si-O**-C_2H_5 asymmetric stretching
955	**Si-O**-H stretching
820, 780	H_3**C-Si**(O) symmetric stretching

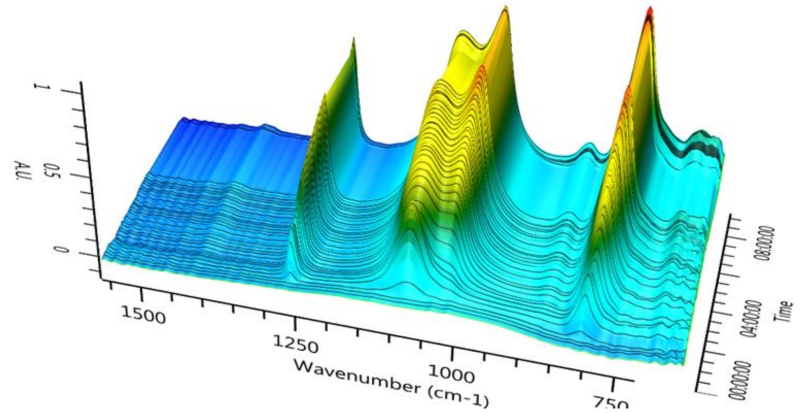

Figure S5. FTIR ATR spectra recorded in the course of cationic emulsion polymerization of D4 alone – a three dimensional picture. Only the range 1400-700 cm-1 where the significant changes were observed is shown.

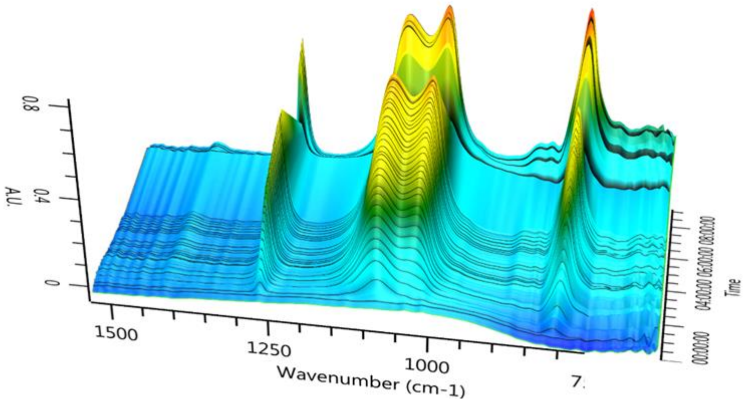

Figure S6. FTIR ATR spectra recorded in the course of cationic emulsion polymerization of D4 in the mixture with VTES – a three dimensional picture. Only the range 1400-700 cm-1 where the significant changes were observed is shown.

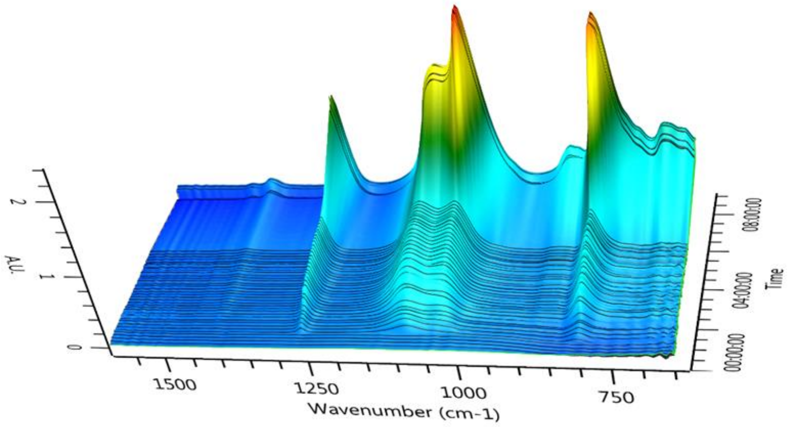

Figure S7. FTIR ATR spectra recorded in the course of cationic emulsion polymerization of D4 in the mixture with VTES and MTES – a three dimensional picture. Only the range 1400-700 cm-1 where the significant changes were observed is shown.

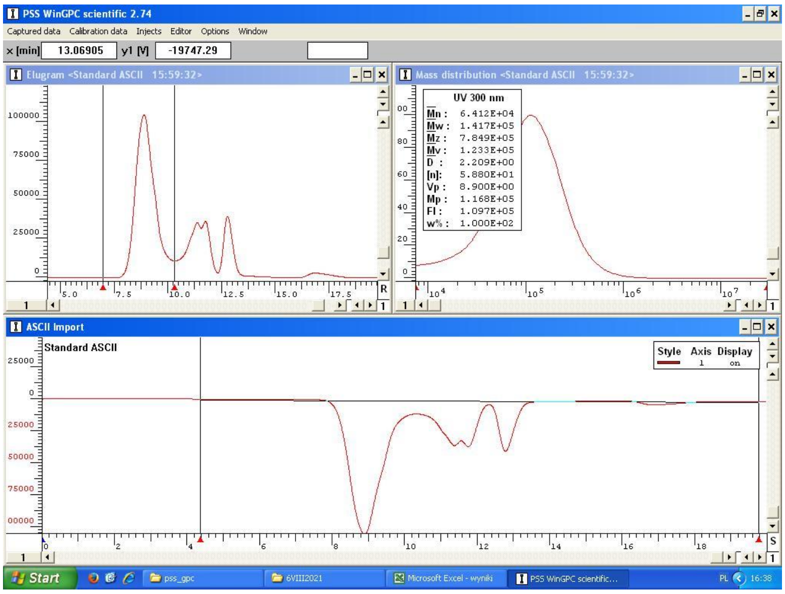

Figure S8. Full GPC chromatogram of the reaction product obtained in cationic emulsion polymerization of D4 alone.

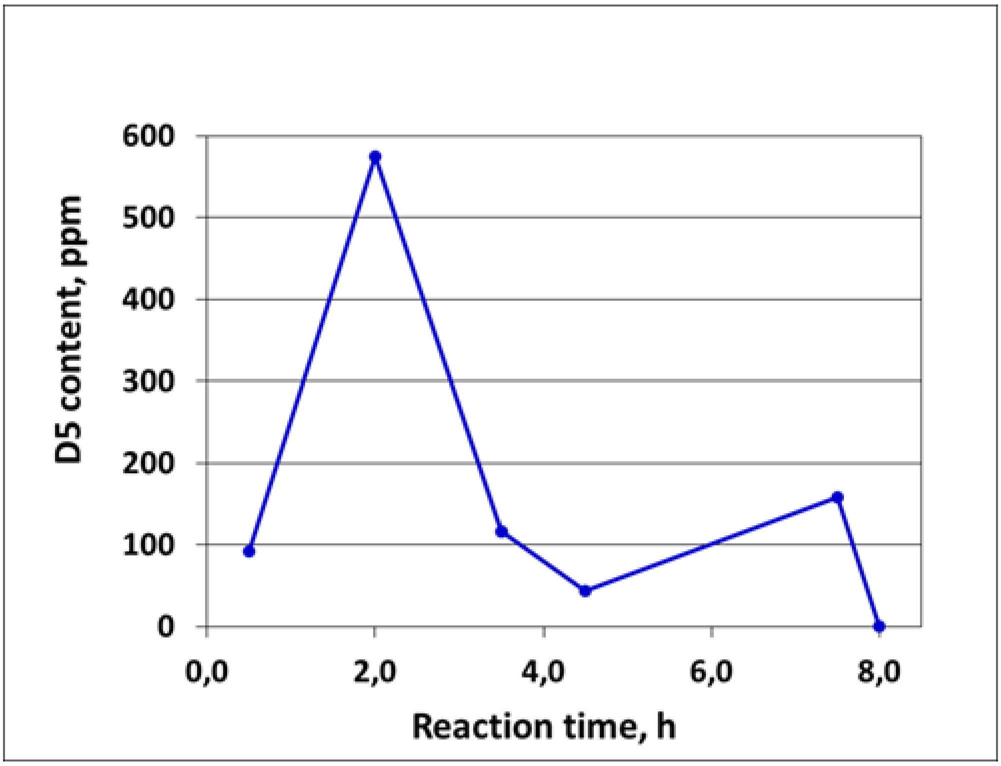

Figure S9. Changes in D5 content in the course of cationic emulsion polymerization of D4 alone as determined by GC.
